# Diagnosis, treatment and follow up of neonatal arrhythmias

**DOI:** 10.5830/CVJA-2014-002

**Published:** 2014-04

**Authors:** Fatih Köksal Binnetoğlu, Kadir Babaoğlu, Gürkan Altun, Gülcan Türker

**Affiliations:** Medical Faculty, Çanakkale Onsekiz Mart University, Çanakkale, Turkey; Department of Paediatric Cardiology, Kocaeli University, Kocaeli, Turkey; Department of Paediatric Cardiology, Kocaeli University, Kocaeli, Turkey; Department of Neonatology, Kocaeli University, Kocaeli, Turkey

**Keywords:** arrhythmia, neonatal, supraventricular tachycardia

## Abstract

**Objective:**

This study aimed to evaluate the aetiology, spectrum, course and outcomes of neonates with arrhythmias observed in a tertiary neonatal intensive care unit from 2007 to 2012.

**Methods:**

Neonates with rhythm problems were included. The results of electrocardiography (ECG), Holter ECG, echocardiography and biochemical analysis were evaluated. The long-term results of follow up were reviewed.

**Results:**

Forty-five patients were male (68%) and 21 (32%) were female. Fifty-five patients (83.3%) were term, 11 (16.6%) were preterm, and 34% were diagnosed in the prenatal period. Twenty cases (30.3%) had congenital heart disease. Twenty-three patients (34.8%) were diagnosed during the foetal period. The most common arrhythmias were supraventricular ectopic beats and supraventricular tachycardia (SVT) at 39.3 and 22.7%, respectively. SVT recurred in five patients after the neonatal period.

**Conclusion:**

Supraventricular ectopic beats and SVT were the most common arrhythmias during the neonatal period. Although the prognosis of arrhythmias in the neonatal period is relatively good, regular monitoring is required.

## Abstract

Arrhythmias are seldom observed in the newborn period and rarely lead to serious consequences. The incidence is about 1% during the neonatal period and 1–3% in late pregnancy.^1^

Long-term tachycardia and bradycardia attacks induced by neonatal arrhythmias may lead to heart failure and hydrops foetalis.^2^ Because they may be a continuation of foetal arrhythmias, newborn arrhythmias are different from those occurring at later ages.^3^ For this reason, the early diagnosis of arrhythmias in the prenatal period is essential for appropriate and optimal treatment in the postnatal period. In this study, we evaluated the type, clinical characteristics, treatment and follow up of newborns with arrhythmias.

## Methods

This study included 66 newborns (45 male, 21 female) diagnosed with arrhythmia in a tertiary hospital between 2007 and 2012. In all cases, sex, birth method, birth weight, week of pregnancy, maternal and gestational diseases, Apgar scores, and haematological and biochemical parameters were recorded. The results of electrocardiography (ECG) with 12 derivations, 24-hour Holter ECG and echocardiography were evaluated. Type, course of arrhythmia, detection time and treatments were analysed retrospectively.

The patients were divided into three groups: irregular heart rhythm (ectopic beats, supraventricular premature beats and ventricular premature beats), bradyarrhythmia [sinus bradycardia, 2:1 atrioventricular (AV) block, complete AV block, long QT syndrome, etc], and tachyarrhythmias (sinus tachycardia, supraventricular tachycardia, ventricular tachycardia). Benign arrhythmias, such as sinus arrhythmia, nodal or junctional rhythms, wandering atrial rhythm, first-degree AV block and Wenckebach block were not included in the study.

The statistical analysis of the results was carried out using the SPSS v13.0 (SPSS Inc., Chicago, IL, USA). Descriptive analyses of the normal variables are given as mean and standard deviation; data with non-normal distribution are given as minimum, maximum and median values.

## Results

Forty-five babies were male (68.2%) and 21 were female (31.8%). The average duration of pregnancy was 38.1 ± 2 weeks (34–41), and the average birth weight was 3 258 ± 508.6 g (2 200–4 500). Approximately 65% of the babies were delivered by Caesarean section; 83.3% were term and 16.6% were preterm (Table 1).

**Table 1:** Demographic features of the study group.

*Demographic features*	*Number (mean ± standard deviation)*
Sex, M/F	45/21
Gestational age (week)	38.1 ± 2 (34–41)
Preterm delivery	11
Term delivery	55
Delivery mode	
Normal	28
Caesarian	38
Apgar score (5 minutes)	8.5 ± 2
Birth weight (g)	3 258 ± 508.6 (2 200–4 500)

The initial clinical presentations were apnoea, poor feeding, irritability, respiratory difficulties and cyanosis. Twenty-one babies were asymptomatic and arrhythmia was diagnosed during routine examination. Thirty patients had benign supraventricular premature beats or ventricular premature beats. Eleven patients had bradyarrhythmia and 25 had tachyarrhythmia (Table 2).

**Table 2:** Distribution and clinical characteristics of patients diagnosed with arrhythmia in the neonatal period

*Arrhythmia type*	*n (%)*	*Accompanying congenital heart disease (n)*	*Accompanying other extracardiac problems*
Irregular heart rhythm (ectopic beats)	30 (45.4)		
Supraventricular premature beats	26 (39.3)	VSDm (1), ASD (1), VSD + ASD (2), ASD + BAV (1), AVSD + HRV (2)	A-V malformation
Ventricular premature beats	4 (6)	ASD (1), VSDm (1)	
Bradyarrhythmias	11 (16.6)		
Sinus bradycardia	3 (4.5)	VSDp (1)	Hypocalcaemia + DiGeorge (1), hypoglycaemia (1)
2:1 AV block	3 (4.5)	TOF (1), atrial isomerism (1)	Sepsis
Complete AV block	3 (4.5)	PDA (1), DILV (1)	Hypothyroidism
Intraventricular block	1 (1.5)	–	Hyperkalaemia (CAH)
Long QT	1 (1.5)	–	–
Tachyarrhythmia	25 (37.8)		
Sinus tachycardia	2 (3)	–	
Supraventricular tachycardia	23 (34.8)		
AVRT*	16 (24.2)	Partial AVSD (1), ASD (3)	Diaphragmatic hernia (1), co-anal atresia (1)
Atrial flutter	5 (7.5)	PDA (1), ASD (1)	Metabolic asidosis, diaphragmatic hernia, hypoglycaemia
MAT	1 (1.5)	–	–
PJRT	1 (1.5)	Dilated cardiyomyopathy (1)	Foetal hydrops
Total	66 (100)		

ASD: atrial septal defect, AV: atrioventricular, A-V: arteriovenous, AVSD: atrioventricular septal defect, AVRT: atrioventricular re-entrant tachycardia, BAV: bicuspid aortic valve, DILV: double-inlet left ventricule, HRV: hypoplastic right ventricule, CAH: congenital adrenal hyperplasia, MAT: multifocalatrial tachycardia, PDA: patent ductus arteriosus, PJRT: permanent junctional reciprocating tachycardia, TOF: tetralogy of Fallot, VSDm: muscular ventricular septal defect, VSDp: perimembranous septal defect. *Three babies had WPW.

Twenty-three patients (34.8%) were diagnosed in the foetal period. Six were premature and 17 were mature. Supraventricular premature beat was the most frequently diagnosed arrhythmia in the foetal period (Table 3). The mother of a baby with complete heart block diagnosed in the foetal period had Sjogren’s disease. Hydrops feotalis and foetal cardiomyopathy occurred in two babies with foetal supraventricular tachycardia (SVT).

**Table 3:** The types and frequency of arrhythmias diagnosed in the foetal period

*Arrhythmia type*	*Number of cases (n = 23)*	*Percent (%)*
Irregular heart rhythm (ectopic beats)	15	65.2
Supraventricular premature beats	13	56.5
Ventricular premature beats	2	2
Bradyarrhythmias	3	13
Sinus bradycardia	1	4.3
2:1 AV block	1	4.3
Complete AV block	1	4.3
Tachyarrhythmia	5	21.7
Supraventricular tachycardia	3	13
Atrial flutter	2	8.7

AV: atrioventricular.

Permanent junctional reciprocating tachycardia (PJRT) was the final diagnosis in patients with foetal cardiomyopathy after delivery. In addition, two babies had atrial flutter. All four of these patients were delivered at 37 weeks. The remaining baby was diagnosed in the 34th week with SVT. Digoxin was given to the mother. However, when rhythm control could not be maintained, sotalol was added and a partial response was achieved. The baby was delivered at 38 weeks. Babies who had complete AV block and 2:1 AV block were not treated in the intrauterine period.

Twenty cases (30.3%) had accompanying congenital heart diseases. Atrial septal defect (ASD) was the most common cardiac pathology. Fifteen babies had ASD, ventricular septal defect or patent ductus arteriosus. Two babies, both of whom were term, had patent ductus arteriosus. Term babies with spontaneously closed ductus arteriosus after the first week were not included in this data. No premature baby subsequently showed patent ductus arteriosus after discharge. In babies with ASD, all defects were small or medium sized. Five babies had complex congenital heart diseases, such as AV septal defect, tetralogy of Fallot, or double-inlet left ventricle.

Twenty-six patients had supraventricular premature beats and four had ventricular premature beats. Anti-arrhythmia treatment was given for two weeks to three months to only six patients who had frequent or couplet–triplet supraventricular premature beats or short-duration SVT attacks determined by 24-hour Holter monitoring. Supraventricular premature beats continued for one year in seven patients.

After discharge, SVT did not occur in any patient with supraventricular premature beats. Patients with ventricular premature beats did not have any anti-arrhythmia treatment, and no additional arrhythmia occurred after the newborn

Eleven babies had bradyarrhythmia, four of whom had sinus bradycardia. One had hypocalcaemia (DiGeorge syndrome), one had hypoglycaemia, and one had long QT syndrome. One baby did not have any pathology. One baby had bradyarrhythmia with intraventricular block. This baby had severe hyperkalaemia (K^+^: 9.4 mEq/l) caused by congenital adrenal hyperplasia.

Three patients had complete AV block and 2:1 AV block. Six patients had AV block – three with complete AV block and one in whom 2:1 AV block later progressed to complete AV block. Four patients with AV block underwent permanent pacemaker implantation.

Two patients with tachyarrhythmia had sinus tachycardia. One of these patients had a persistent heart rate of 180–190 beats/min and was given short-term treatment with a beta-blocker, which was discontinued after the newborn period. The other baby did not have any treatment.

Twenty-three babies had SVT: five had atrial flutter (AF), one had PJRT, one had multifocal atrial tachycardia (MAT), and the rest had atrioventricular re-entrant tachycardia (AVRT). No baby had atrioventricular nodal re-entrant tachycardia (AVNRT). Sinus rhythm was achieved with cardioversion in four patients with AF and in one patient with amiodarone infusion. In babies with SVT without AF, six were treated with adenosine, four were treated with amiodarone and application of ice to the face, two were treated with ice initially and then with adenosine, and two were treated with digoxin. Three babies with SVT had Wolf–Parkinson–White (WPW) syndrome. Digoxin or propranolol prophylaxis was given to all patients with SVT and AF.

The average duration of follow up after the newborn period for all patients was 15 months (minimum three months, maximum six years). Twenty-three patients with SVT and three with frequent supraventricular premature beats continued anti-arrhythmia treatment after discharge (beta-blockers or digoxin). AF did not recur in any patients. Five patients with SVT had recurrence after the newborn period. In these patients, rhythm and speed control was achieved by treatment with sotalol. Patients with PJRT and multifocal atrial tachycardia required anti-arrhythmia treatment after the age of one year. Three patients with WPW syndrome continued to take propranolol prophylaxis.

Four patients died in the newborn period. One baby with diaphragmatic hernia and one with co-anal atresia died because of respiratory problems after SVT treatment. The other baby had supraventricular premature beats diagnosed in the foetal period. In spite of spontaneously resolved supraventricular premature beats on the second postnatal day, this patient died because of metabolic disease. The remaining baby had tetralogy of Fallot 2:1 AV block, which progressed to complete AV block. This baby died because of sepsis after a pacemaker implantation. Apart from these four babies, no other patients died during the follow-up period.

## Discussion

The incidence of arrhythmia in the newborn period has been reported to be about 1%.^4^ Most of these arrhythmias are asymptomatic and rarely life-threatening.

Various studies have identified that 15.3% of arrhythmic newborns have congenital heart disease. Atrial arrhythmias in particular are reported more frequently in newborns with congenital heart disease.^5^,^6^ In a study of 21 arrhythmic newborns, Satar *et al.*^7^ found a congenital heart disease rate of 38%, while Canpolat *et al.*^8^ reported a rate of 23.1%. Of our cases, 30.3% had congenital heart disease. Although in our study group the most frequent accompanying pathology was ASD, Satar *et al.*^7^ found that patent ductus arteriosus was the most frequent pathology.

Ventricular and supraventricular premature beats are generally self-limiting, benign arrhythmias. Examination of healthy newborns before discharge determined a 1% frequency of premature beats. Premature beats may be secondary to metabolic and biochemical abnormalities and hypoxia; however, the majority have no clear underlying pathology.^9^

The prognosis for premature beats is generally very good, and most of them disappear after the first months of life. Of our patients, 39.3% had supraventricular premature beats and 6% had ventricular premature beats. Patients with ventricular premature beats did not have arrhythmia after the newborn period. Seven patients with supraventricular premature beats had persistent arrhythmia beyond one year, but none of them progressed to SVT. In a study by Poddar *et al.*,^10^ premature beats lasted up to early childhood and spontaneously resolved without complications in three of nine arrhythmic newborns. Our results were compatible with the literature.

Congenital complete AV block had an observed rate of 1/15 000–20 000 for live births.^11^ Generally, it is secondary to structural cardiac defects or maternal systemic lupus erythematosus.^12^ Complete AV block with severe bradycardia leading to low cardiac output may result in heart failure. Symptomatic complete AV block and asymptomatic block with heart rate below 55 beats/min, accompanied by wide QRS escape rhythm or accompanying cardiomegaly, are indications for emergency pacemaker implantation.^11^

Canpolat *et al.*^8^ identified four patients (15.4%) with AV block, all of whom were diagnosed in the prenatal period. Only two had mothers with lupus or Sjogren’s disease. In our study, four of six patients with AV block underwent pacemaker implantation, and two were diagnosed in the prenatal period.

While SVT is the most frequently observed type of tachycardia in the newborn period, it can cause postnatal irritability, feeding difficulties, tachypnoea, tachycardia and heart failure in the antenatal period. Many newborns can tolerate the first hours of SVT well, but if SVT continues longer than 6–12 hours, heart failure caused by stroke volume reduction may develop.^13^ Fifteen per cent of patients have a history of sepsis and medication.^14^ Additionally, babies with cardiac anomalies, such as Ebstein anomaly, transposition of the great arteries, and single ventricle are known to be at risk for SVT.^11^

In our study, SVT was the second most frequent arrhythmia (22.7%). This result was compatible with the literature. However, this rate is lower than the SVT rate found in the study by Satar *et al.*^7^ In a similar study,^15^ eight patients had major cardiac pathologies, such as Ebstein anomaly, AV septal defect, ventricular septal defect and tricuspid atresia. However, none of our patients had complex cardiac pathologies.

AV nodal re-entrant tachycardia is rarely seen in the newborn and toddler periods. In this period, AV re-entrant tachycardia is more frequent. Naheed *et al.*^16^ studied 30 foetuses with SVT and did not detect AV nodal re-entrant tachycardia in the postnatal period. In a study by Ko *et al.*,^17^ only three of 137 (2.1%) had AV nodal re-entrant tachycardia. No patient in our study had AV nodal re-entrant tachycardia. In our cases with SVT, AV re-entrant tachycardia was the most frequent mechanism of tachycardia.

In WPW syndrome, apart from the normal communicating paths, accessory pathways link the atrium and ventricles. According to Lupoglazoff and Denjoy, 70% of babies less than three months old with SVT have WPW pattern.^18^ In a study by Gillijam *et al.*,^15^ the rate of WPW syndrome was 34%. Kundak *et al.*^19^ studied 55 newborns with malignant rhythms; 22 had SVT and six had WPW syndrome.

In our study, three patients had WPW syndrome. In another study,^20^ 90 babies had WPW syndrome, one-third of whom had repeating SVT episodes after the age of one year. In this study, the majority of babies were given prophylactic anti-arrhythmia treatment. In our study, all three babies with WPW syndrome were given propranolol prophylaxis. These babies did not have an SVT episode during the long-term follow-up period, even after the newborn period.

Gillijam *et al.*^15^ reported that most babies with a history of SVT had taken anti-arrhythmia treatment for 6–12 months after the last SVT episode. In our study, only five patients with SVT had repeating episodes beyond the newborn period, and it persisted beyond the age of one year in only two patients, which is compatible with the literature.

Ventricular tachyarrhythmia, while rare in the newborn period, generally develops secondary to metabolic anomalies, such as hyperkalaemia, hypoglycaemia, metabolic acidosis and hypoxia. It resolves quickly with treatment of the underlying cause.^21^,^22^ In a study by Kundak *et al.*^19^ six patients had ventricular tachycardia, and three had SVT accompanied by ventricular tachycardia. In our study, none of the patients with metabolic acidosis, sepsis and hypoglycaemia had ventricular tachycardia.

A proportion of newborn arrhythmias are a continuation of arrhythmias that began in the foetal period and extend into the postnatal period. Fifty per cent of patients who had isolated supraventricular premature beats were referred in the foetal period because of irregular heart rhythm. Less than 10% of foetal arrhythmias are in the form of continuous tachyarrhythmia and bradyarrhythmia.^23^ The most common foetal tachyarrhythmias are tachycardia with frequent supraventricular premature beats, SVT, AF and, rarely, ventricular tachycardia.

Foetal supraventricular premature beats have a very good prognosis but 0.4% of cases may advance to life-threatening tachyarrhythmia.^24^ Foetal AF is mostly associated with structural cardiac anomalies; 7–43% of them progress to hydrops.^25^ The most important cause of foetal bradycardia is congenital AV block. Fifty per cent of foetal bradycardia foetuses have mothers with connective tissue diseases, such as lupus and Sjogren’s disease. The remaining 50% have underlying complex cardiac anomalies.^26^

In our study, 23 (34%) newborns had arrhythmias diagnosed in the foetal period. Three of these had SVT, and two had AF. One had complete AV block, and one had 2:1 AV block. Hydrops developed in one patient with foetal SVT.

## Conclusion

Although the frequency of arrhythmias in the newborn period is not high, supraventricular premature beats and SVT are the most frequently observed arrhythmias in this period. Diagnosis of arrhythmias in the prenatal period is essential for appropriate and optimal treatment in the postnatal period. Although the longterm prognosis for newborn arrhythmias is very good, patients should be monitored at appropriate intervals.
